# Fluoroscopic‐Guided Removal of a Retained Bullet From the Chest Under Local Anesthesia

**DOI:** 10.1155/cris/4631801

**Published:** 2026-03-23

**Authors:** Murad Alahmad, Omran Almokdad, Ibrahim Taha, Ayman El-Menyar, Hassan Al-Thani, Sohaib Zoghoul, Ali Barah, Ruben Peralta

**Affiliations:** ^1^ Department of Trauma Surgery, Hamad Medical Corporation, Doha, Qatar, hamad.qa; ^2^ Department of Radiology, Hamad Medical Corporation, Doha, Qatar, hamad.qa; ^3^ Department of Trauma Surgery, Clinical Research, Hamad Medical Corporation, Doha, Qatar, hamad.qa; ^4^ Clinical Medicine, Weill Cornell Medicine, Doha, Qatar, cornell.edu; ^5^ Department of Surgery, Universidad Nacional Pedro Henriquez Urena, 10100, Santo Domingo, Dominican Republic, unphu.edu.do

**Keywords:** firearm injury, fluoroscopy, psychology, retained bullet

## Abstract

Firearm injuries (FAIs) are a significant public health concern, often resulting in retained bullets (RBs) that pose management challenges. We present a 24‐year‐old male with a retained subcutaneous chest wall bullet ~2 cm lateral to the midclavicular line, causing persistent psychological distress and functional impairment. Initial attempts at bullet removal under local anesthesia failed, leading to nonoperative management. However, the patient’s ongoing anxiety, fear of death, and inability to work necessitated reconsideration of treatment. Fluoroscopy‐guided extraction under local anesthesia enabled precise localization of the RB and facilitated targeted dissection, avoiding blind exploration, minimizing tissue trauma, and eliminating the need for general anesthesia. The procedure was completed successfully with resolution of symptoms. The case underscores the importance of integrating psychological considerations into RB management and highlights fluoroscopy‐guided extraction as a safe, minimally invasive, and effective technique for bullet removal, particularly in anatomically challenging cases or after failed blind exploration.

## 1. Introduction

Firearm injuries (FAIs) pose a significant public health concern worldwide, contributing to substantial morbidity and mortality [1]. The increased accessibility of firearms to civilians has increased the incidence of FAI. It is estimated that FAI accounts for 20% of all injury‐related deaths and is responsible for 105,000 injuries annually in the United States [2]. As the incidence of gunshot victims increases, the number of patients with retained bullets (RBs) or their fragments also increases. Beyond the immediate physical harm, survivors frequently experience substantial psychological sequelae, including anxiety, depression, posttraumatic stress symptoms, and reduced quality of life, which may persist long after the initial injury [3]. Herein, we present a case of an RB in the chest, which was removed using fluoroscopy guidance under local anesthesia due to persistent patient psychological stress.

## 2. Case Presentation

A 24‐year‐old male construction worker presented to the trauma center following an unusual incident. He was brought to a Level I urban trauma center (Hamad Medical Corporation). While working, he had witnessed a gunshot to the chest. A nearby colleague called for an ambulance, and he was transported to the hospital. Upon arrival, the patient was alert and stable, with a Glasgow Coma Scale (GCS) score of 15. Physical examination revealed bilateral air entry and no evidence of active bleeding. A single penetrating wound measuring ~2 cm in diameter was identified on the anterior chest wall, located about 2 cm lateral to the right midclavicular line. The wound followed a tangential trajectory, and no foreign body was palpable.

**Figure 1:**
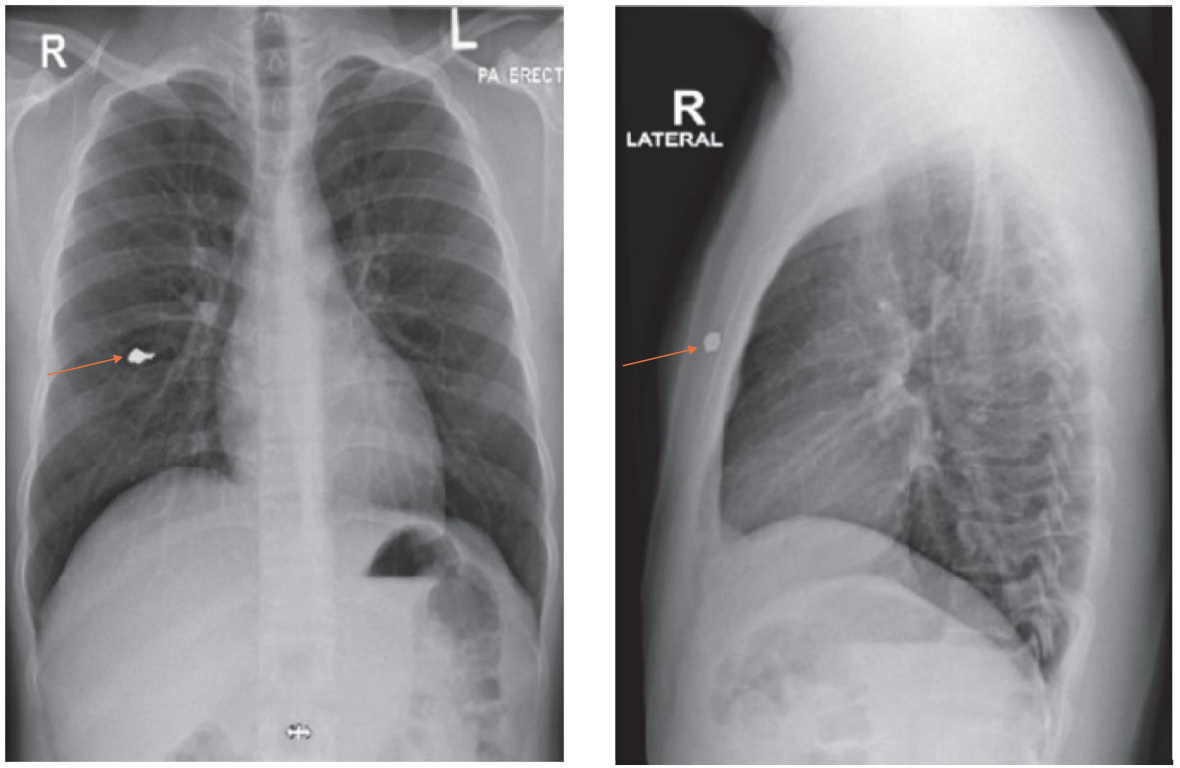
Chest X‐ray showing retained bullet.

**Figure 2:**
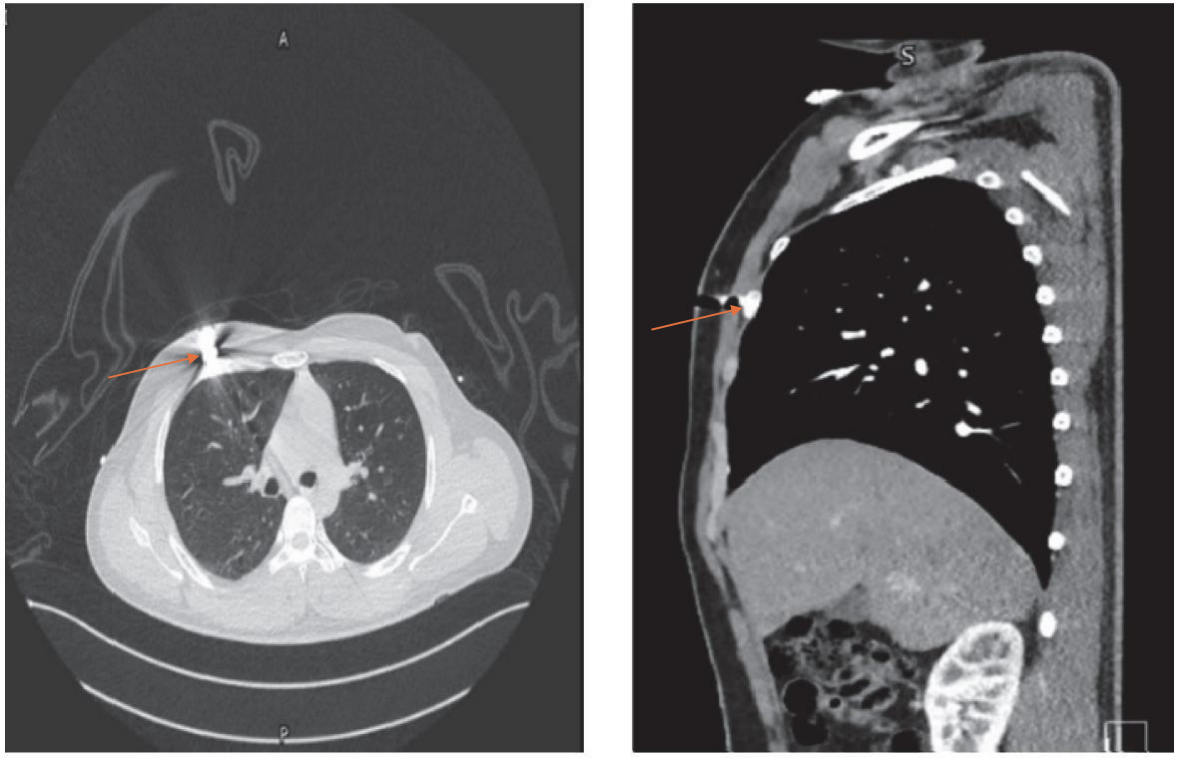
Chest CT scan showing retained bullet.

Initial imaging included portable anterior–posterior and lateral chest X‐rays (Figure 1), which revealed a lodged bullet that had not penetrated the thoracic cavity. A subsequent CT scan confirmed these findings (Figure 2) with bilateral air entry and no evidence of active bleeding. A penetrating wound, ~7 mm in diameter, was noted on his chest, but it was not actively bleeding, and no foreign body could be palpated. Initial imaging included portable anterior–posterior and lateral chest X‐rays (Figure 1), which revealed a lodged bullet that had not penetrated the thoracic cavity. A subsequent CT scan confirmed these findings (Figure 2). CT imaging demonstrated that the bullet was lodged deep to the pectoralis major muscle without intrathoracic or major vascular involvement. Bedside local wound exploration under local anesthesia was attempted, but extraction failed. The attempt was unsuccessful because the bullet was deeply embedded beneath the pectoralis major muscle along a tangential tract, making it nonpalpable and difficult to localize, and blind dissection was deemed unsafe. To avoid unnecessary tissue damage, the procedure was aborted. The patient was reassured that the bullet posed no immediate risk and that removal might require general anesthesia and a larger incision, which has its risks. He received a single dose of antibiotics and a tetanus booster and was discharged with pain medication and follow‐up instructions. Despite the reassurance, the patient returned to the clinic and emergency department (ED) on multiple occasions over the next 3 months, reporting persistent discomfort and anxiety about the bullet’s potential migration and death. He expressed difficulty sleeping and was referred to a psychologist for evaluation. During his final follow‐up, he reported being unable to work and sleep due to stress and fear of death and insisted on the removal of the foreign body. Although RBs do not always require removal, extraction in this case was considered due to persistent psychological distress affecting his quality of life and functional status. After discussing his options, a decision was made to proceed with extraction.

**Figure 3:**
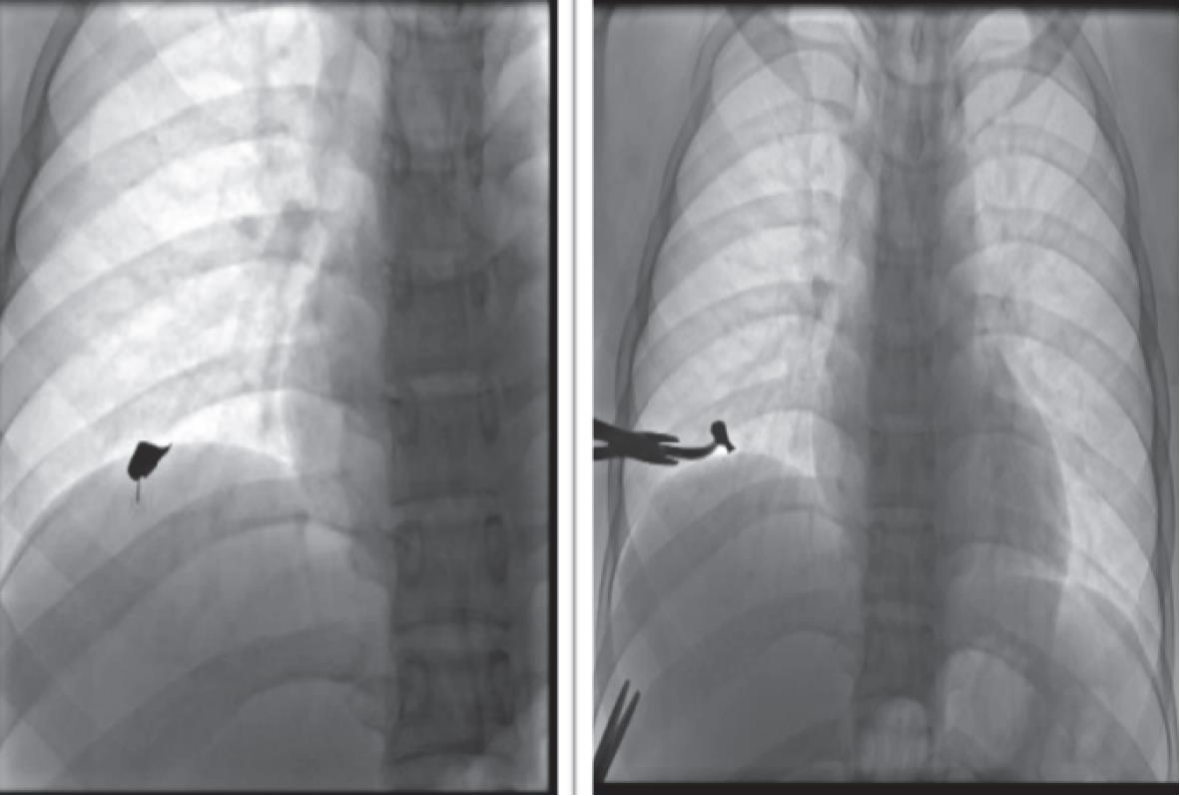
Retained bullet during guided extraction.

**Figure 4:**
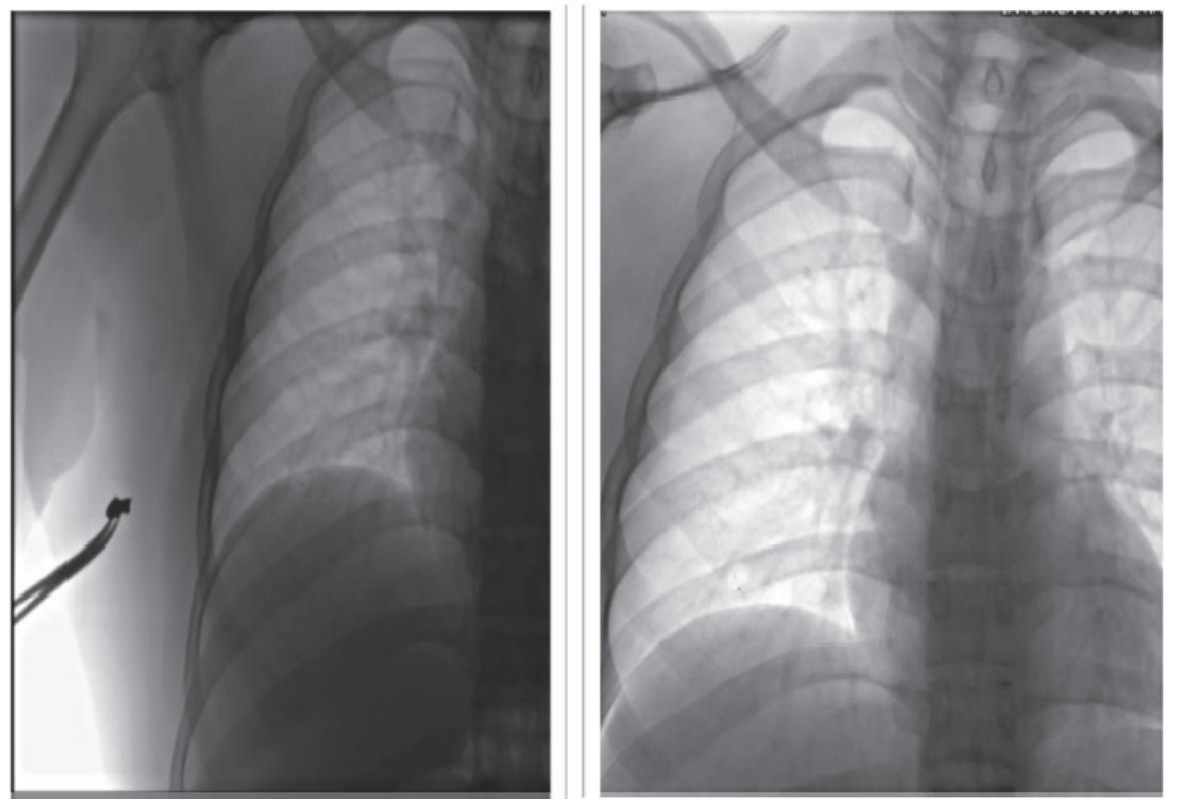
Chest X‐ray after bullet extraction.

Over 3 months, the bullet is expected to become encapsulated in fibrotic tissue, complicating removal and increasing the need for a larger incision, extensive dissection, and potentially general anesthesia. The procedure was planned as a same‐day admission in the interventional radiology suite, under local anesthesia with fluoroscopic guidance, to minimize these risks. Under aseptic conditions, local anesthesia was administered, and the bullet location was marked with a needle for incision planning. A 1.5 cm incision was made along the previous scar, and guided dissection was used to locate and successfully remove the bullet (Figures 3 and 4).

**Figure 5:**
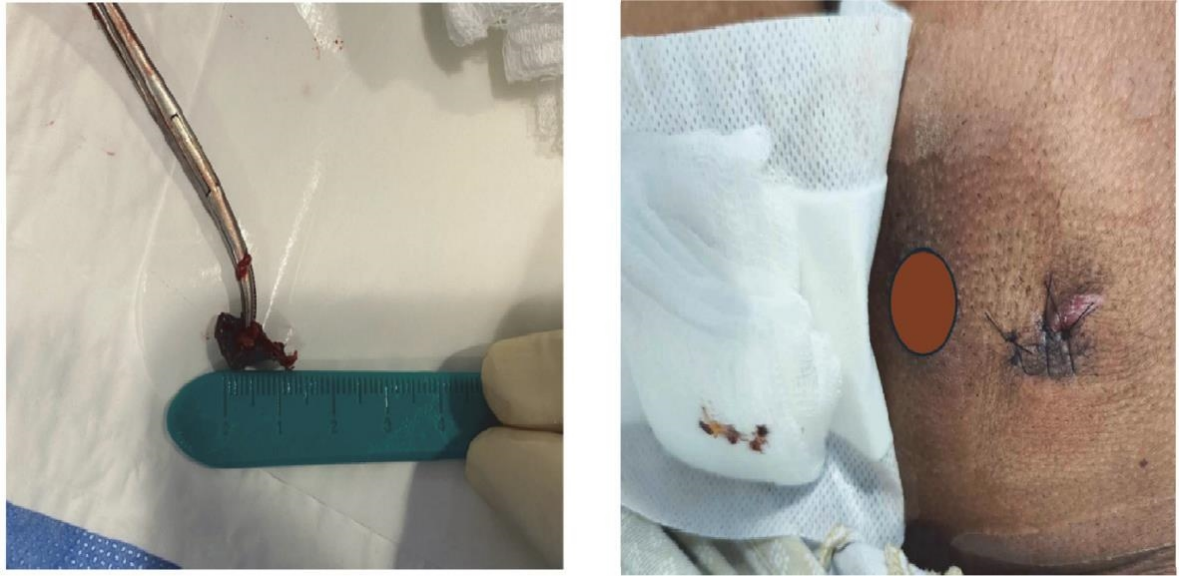
Incision and fragment of the bullet.

The wound was closed with three stitches using Ethilon 3‐0 (Figure 5). The procedure took 20 min, and the patient was discharged the same day. At follow‐up, the patient reported significant improvement, with the wound healing well and high satisfaction with the outcome.

## 3. Discussion

The management of RB has long been a topic of debate in trauma care, and guidelines for treatment are scarce. Neither the Advanced Trauma Life Support (ATLS) guidelines nor the Eastern Association for the Surgery of Trauma (EAST) provide formal recommendations or standardized protocols for the extraction of RBs, leading to significant variability in clinical practice. A 2022 survey found that only 14.5% of participating surgeons reported that their hospital had a bullet removal policy [4]. Although many clinicians support routine bullet removal, its actual necessity depends on the injury’s severity, the extent of tissue involvement, and the location. In many cases, removal is unnecessary and carries risks such as infection, neurovascular damage, and bleeding. Thus, clinical judgment remains paramount [5]. Highlighting this gap in established trauma guidelines underscores the novelty and relevance of approaches such as fluoroscopy‐guided extraction, particularly in cases where psychological distress is a key consideration.

While many RBs remain asymptomatic and may not necessitate removal, there are specific indications for extraction. A study by Dienstknecht et al. [6] suggested that bullets in joints, cerebrospinal fluid, or the eye globe should be removed. Other indications for removal include bullets in blood vessels that could migrate, those causing lead toxicity, palpable bullets that can be easily extracted, and cases involving medico‐legal considerations. RB is a common occurrence following FAIs; however, limited research has been conducted on its diverse effects, particularly their psychological impact on survivors [7]. The mental health implications of RB often go underappreciated in trauma care. Routinely addressing RB during outpatient follow‐ups in FAI patients has been associated with a nearly doubled likelihood of psychological illness screening and diagnosis, emphasizing the significant mental health burden these injuries may pose [4, 8]. Survivors of FAIs may experience a significant psychological burden that influences recovery, including fear of complications, perceived threat to life, and social or occupational limitations. These concerns contribute to ongoing healthcare utilization and highlight gaps in mental health evaluation and follow‐up after injury, underscoring the importance of integrating mental health assessment and referral into routine postinjury care [4, 9]. In our case, mental health considerations played a pivotal role in the decision to pursue extraction. Despite initial reassurance, the patient experienced severe anxiety over the possibility of bullet migration and life‐threatening complications, which profoundly impacted his mental well‐being and daily life. An initial attempt at extraction under local anesthesia in the trauma room was unsuccessful, and was advocated for nonoperative management to avoid unnecessary harm. However, the patient’s ongoing anxiety, persistent pain, inability to work, and fear of death ultimately justified proceeding with extraction.

Fluoroscopy, an X‐ray‐based imaging technique, is becoming increasingly popular as a minimally invasive method for accurately locating and removing retained foreign bodies [10]. Unlike traditional approaches that may require larger incisions or general anesthesia, fluoroscopic guidance provides real‐time imaging that facilitates precise localization, reduces the need for extensive dissection, minimizes tissue trauma, and lowers the risk of complications. It is particularly valuable when bullets are difficult to palpate, located within soft tissue planes, or after unsuccessful blind exploration, as it enables targeted dissection while minimizing operative morbidity [11]. In our case, where the RB was difficult to palpate and previous attempts at extraction had failed, fluoroscopy provided a safer, more efficient alternative to conventional surgery. Fluoroscopy‐guided removal of RB has proven to be a safe, minimally invasive, and effective alternative to traditional surgery, particularly in cases with previous failed extraction attempts or difficult localization. Given its potential advantages, fluoroscopy‐guided extraction should be further considered in RB management, as it reduces complications and improves patient outcomes. Future research should prioritize developing standardized protocols that clearly differentiate indications for RB removal based on psychological versus anatomical criteria. Prospective cohort studies comparing patient outcomes for psychological indications (such as persistent distress or functional impairment) versus anatomic indications (such as risk of migration or tissue involvement) would help delineate best practices and inform multidisciplinary guidelines. Additionally, establishing the role of fluoroscopy‐guided extraction across these varied indications could galvanize effective collaboration and advance the evidence base for optimal patient care.

## 4. Conclusion

This case highlights the importance of addressing both the physical and psychological factors in managing RBs. Persistent psychological distress, such as anxiety and fear of complications, can significantly impact a patient’s well‐being, functional status, and quality of life. The psychological burden following FAI is well documented in the broader literature, and this case reinforces the need to recognize mental health considerations as a legitimate indication for RB removal in selected patients.

## Author Contributions

All authors contributed substantially to this manuscript.

## Funding

The authors have nothing to report.

## Disclosure

All authors read and approved the manuscript for submission.

## Ethics Statement

Ethical approval was obtained from the institutional review board (MRC‐04‐24‐897) at the Medical Research Center, Hamad Medical Corporation (HMC), Doha, Qatar. The Medical Research Center (MRC) granted a waiver of consent for publication, provided that no photo or personal identifiers were included.

## Conflicts of Interest

The authors declare no conflicts of interest.

## Data Availability

Data sharing is not applicable to this article as no datasets were generated or analyzed during the current study.
